# Antitumor effects of regorafenib and sorafenib in preclinical models of hepatocellular carcinoma

**DOI:** 10.18632/oncotarget.22334

**Published:** 2017-11-06

**Authors:** Maria Kissel, Sandra Berndt, Lukas Fiebig, Simon Kling, Qunsheng Ji, Qingyang Gu, Tina Lang, Frank-Thorsten Hafner, Michael Teufel, Dieter Zopf

**Affiliations:** ^1^ Drug Discovery, Bayer AG, Wuppertal, Germany; ^2^ Drug Discovery, Bayer AG, Berlin, Germany; ^3^ Biochemistry, NMI Natural and Medicinal Sciences Institute, University of Tübingen, Reutlingen, Germany; ^4^ Research Service Division, Oncology & Immunology Unit, WuXi AppTec Co. Ltd., Shanghai, China; ^5^ Research & Clinical Sciences Statistics, Bayer AG, Berlin, Germany; ^6^ Translational Medicine Oncology, Bayer HealthCare Pharmaceuticals, Whippany, NJ, USA

**Keywords:** regorafenib, sorafenib, HCC, antitumor activity, preclinical pharmacology

## Abstract

The purpose of this study was to investigate the antitumor activity of regorafenib and sorafenib in preclinical models of HCC and to assess their mechanism of action by associated changes in protein expression in a HCC-PDX mouse model. Both drugs were administered orally once daily at 10 mg/kg (regorafenib) or 30 mg/kg (sorafenib), which recapitulate the human exposure at the maximally tolerated dose in mice.

In a H129 hepatoma model, survival times differed significantly between regorafenib versus vehicle (p=0.0269; median survival times 36 vs 27 days), but not between sorafenib versus vehicle (p=0.1961; 33 vs 28 days). Effects on tumor growth were assessed in 10 patient-derived HCC xenograft (HCC-PDX) models. Significant tumor growth inhibition was observed in 8/10 models with regorafenib and 7/10 with sorafenib; in four models, superior response was observed with regorafenib versus sorafenib which was deemed not to be due to lower sorafenib exposure. Bead-based multiplex western blot analysis was performed with total protein lysates from drug- and vehicle-treated HCC-PDX xenografts. Protein expression was substantially different in regorafenib- and sorafenib-treated samples compared with vehicle. The pattern of upregulated proteins was similar with both drugs and indicates an activated RAF/MEK/ERK pathway, but more proteins were downregulated with sorafenib versus regorafenib. Overall, both regorafenib and sorafenib were effective in mouse models of HCC, although several cases showed better regorafenib activity which may explain the observed efficacy of regorafenib in sorafenib-refractory patients.

## INTRODUCTION

Hepatocellular carcinoma (HCC) is the most common primary malignancy of the liver, with more than 500,000 new cases diagnosed per year worldwide [1]. HCC is the second leading cause of cancer-related mortality across the world. Although surgery is potentially curative in some cases, most patients are diagnosed with advanced HCC for which there are limited treatment options available [2]. The pathogenesis of HCC involves changes in several signaling cascades including those involved in cell proliferation and angiogenesis [3]. Hence, there is a strong rationale for using targeted agents such as multikinase inhibitors (MKIs) for the treatment of HCC.

Sorafenib was the first MKI to demonstrate a significant improvement in clinical outcomes in HCC, and has demonstrated activity against a number of kinase targets including RAF-1, B-RAF, KIT, RET, vascular endothelial growth factor receptors (VEGFRs) 1–3, and platelet-derived growth factor receptor (PDGFR) [4, 5]. In two phase 3 trials (SHARP and SHARP-AP), sorafenib was significantly associated with increased overall survival (OS) in patients with unresectable HCC (uHCC) [6, 7]. In the SHARP trial, sorafenib improved OS in patients with uHCC with a median OS of 10.7 months with sorafenib versus 7.9 months with placebo (hazard ratio [HR] 0.69, 95% confidence interval [CI] 0.55, 0.87; p<0.001) [6]. In the SHARP-AP trial, the median OS was 6.5 months with sorafenib versus 4.2 months with placebo (HR 0.68, 95% CI 0.50, 0.93; p=0.014) [7]; following these results, sorafenib was approved as first-line treatment for uHCC at 800 mg/day [8, 9].

Regorafenib is a different oral MKI that potently blocks multiple protein kinases involved in tumor angiogenesis (VEGFRs 1–3, TIE2), oncogenesis (KIT, RET, RAF-1, BRAF), metastasis (VEGFR3, PDGFR, fibroblast growth factor receptor), and tumor immunity (CSF1R) [10, 11]. In the phase 3 RESORCE trial, regorafenib was significantly associated with improved OS compared with placebo in patients with uHCC who progressed on sorafenib treatment, with a median OS of 10.6 months with regorafenib versus 7.8 months with placebo (HR 0.63, 95% CI 0.50, 0.79; p<0.0001) [12]. The survival benefit provided by the sequential use of regorafenib after progression on sorafenib suggests that, despite their similarities, regorafenib and sorafenib have important differences in their mechanisms of action.

The purpose of the present study was to investigate the antitumor activity of regorafenib and sorafenib, in various mouse models of HCC, and to analyze associated pharmacodynamic changes in the expression of functionally relevant proteins in order to better understand potential differences in their mechanisms of action.

## RESULTS

### Syngenic orthotopic H129 mouse model

**Figure 1:**
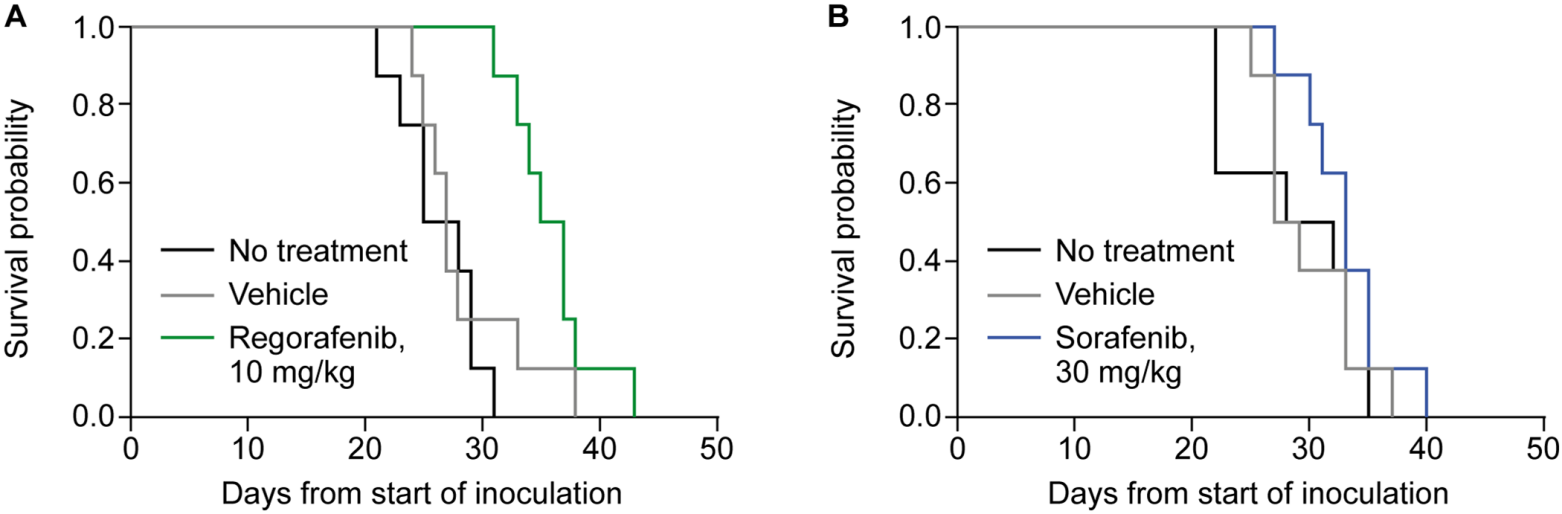
Survival of mice carrying orthotopic H129 liver tumors without treatment or treated with vehicle, regorafenib, or sorafenib Treatments with regorafenib **(A)** and sorafenib **(B)** were performed in separate studies. The survival times are measured in days.

The antitumor activities of regorafenib and sorafenib were assessed in a H129 hepatoma model orthotopically transplanted into the liver of syngenic mice. Treatment was initiated 4–5 days after tumor implantation with once-daily oral regorafenib 10 mg/kg, vehicle solution, or the mice were left untreated (n=8 per group). The log-rank test showed significantly different survival time distributions between regorafenib-treated and vehicle-treated animals (p=0.0269; median survival times: 36 days for regorafenib-treated animals vs 27 days for vehicle-treated animals) (Figure 1A and Table 1). In a similar study, mice were treated with once-daily oral sorafenib 30 mg/kg, vehicle solution, or were left untreated (n=8 per group). Sorafenib treatment did not result in a significant improvement in survival compared with vehicle-treated animals, with survival times of 33 days and 28 days, respectively (log-rank test p=0.1961) (Figure 1B and Table 1). In both studies, survival times of untreated animals were similar to vehicle-treated animals (Table 1). Both compounds were well tolerated as assessed by predefined parameters (see Material and Methods) and no drug-related deaths occurred.

**Table 1:** Median survival times (days) of mice carrying orthotopic H129 liver tumors treated with either regorafenib 10 mg/kg/day or sorafenib 30 mg/kg/day

Treatment	Median survival, days (95% CI)	
	Regorafenib	Sorafenib
**None**	27 (21, 29)	30 (22, 35)
**Vehicle**	27 (24, 33)	28 (25, 33)
**Compound**	36 (31, 38)	33 (27, 35)

Data are from two separate studies. CI, confidence interval.

### Patient-derived HCC xenograft mouse model

**Figure 2:**
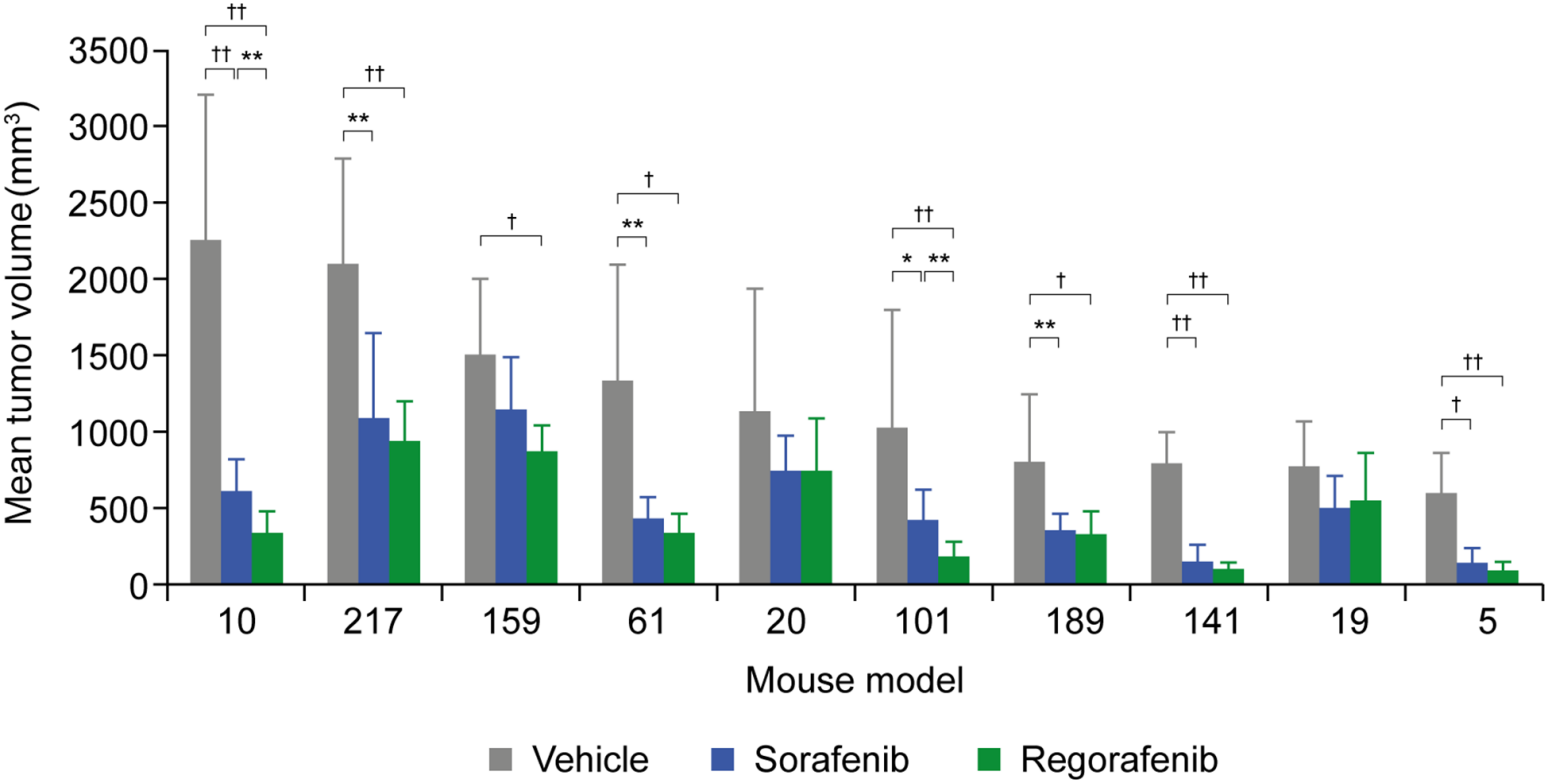
Tumor growth inhibition of 10 HCC-PDX models treated with vehicle, regorafenib, or sorafenib Models are sorted according to mean tumor volumes of the vehicle-treated groups. Detailed numbers are provided in Supplementary Tables 1 and 2. ^*^p<0.05; ^**^p<0.01; ^†^p<0.001; ^††^p<0.0001; no label = not significant; error bars indicate standard deviation. HCC-PDX, patient-derived hepatocellular carcinoma xenograft.

**Figure 3:**
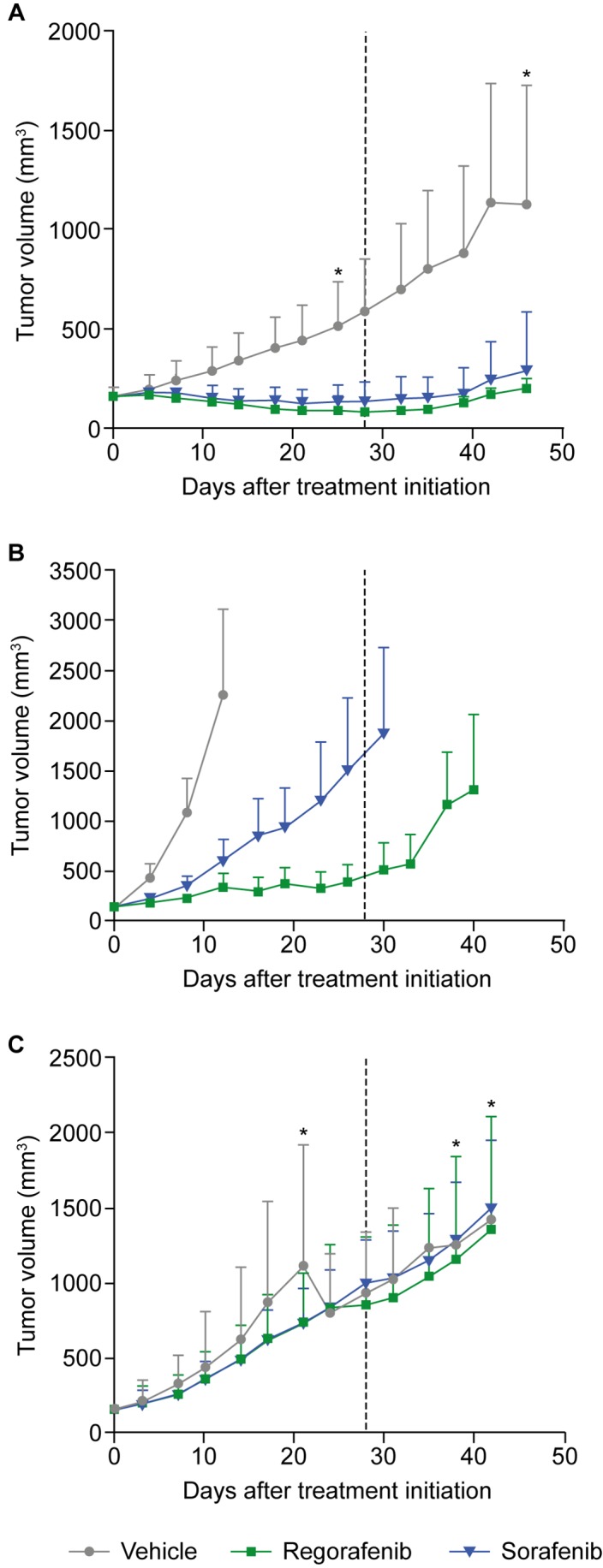
Tumor growth curves of three HCC-PDX models representing different responses to regorafenib and sorafenib treatment Tumor-bearing mice were treated orally for 28 days (dotted line) with regorafenib at 10 mg/kg, sorafenib at 30 mg/kg, or with vehicle. Asterisks mark time points when animals were lost/sacrificed. **(A)** Responder (model 5); **(B)** differential responder (model 10); **(C)** non-responder (model 20). Error bars indicate standard deviations. HCC-PDX, patient-derived hepatocellular carcinoma xenograft.

The effects of regorafenib and sorafenib on tumor growth were further assessed in 10 patient-derived HCC xenograft (HCC-PDX) models (Supplementary Table 1). As before, mice were treated with regorafenib at an initial dose of 10 mg/kg/day, sorafenib at an initial dose of 30 mg/kg/day, or vehicle for a period of 28 days (unless the mice had to be sacrificed because the xenografts exceeded the size of 2000 mm^3^). In all cases, except for models 189 and 217, tumor growth was monitored for an extended period of time after treatment cessation. Tumor growth inhibition (TGI) was assessed at two time points in the study: at the end of the treatment period or when the vehicle group was terminated, and at the end of the study, which covers an observation period without treatment for tumor regrowth. For the first time point, tumor volumes of treatment and vehicle groups were compared (T/C ratio); for the second time point, tumor volumes at the beginning of treatment were compared to those at study termination within the same group (relative tumor volume [RTV]). The extent of TGI varied across the different models, with remarkable responses in models 5 and 141 for both compounds and only marginal responses in models 19 and 20 with T/C ratios >0.6 (Supplementary Table 2). Significant TGI with regorafenib was observed in eight of the 10 models (models 5, 10, 61, 101, 141, 159, 189, 217) and in seven out of 10 for sorafenib (same as regorafenib, except for model 159) (Figure 2 and Supplementary Table 2). In model 19, significant inhibitory effects only became apparent upon RTV evaluation (Supplementary Table 2), whereas model 20 did not show a significant response to either drug (Figures 2 and 3; Supplementary Table 2). A superior response to regorafenib versus sorafenib was observed in models 10 (p=0.0021) and 101 (p=0.0023) when evaluated at the end of treatment; the superior response of regorafenib extended to models 61 (p=0.0081) and 141 (p=0.0474) when RTVs were analyzed at the end of the study (Figures 2 and 3; Supplementary Table 2). Various response types are depicted in more detail by the tumor growth curves of models 5 (responder), 10 (mixed response), and 20 (non-responder) (Figure 3). Based on initial analyses, no association was observed between TGI and characteristics of mice tumor xenograft models; for example, effective inhibition of tumor growth was observed in both fast-growing (model 10) and slow-growing (model 5) xenografts (Figure 2). However, this finding should be interpreted with caution owing to the small number of models.

Treatment was generally well tolerated. Body weight loss did not exceed 10% and no deaths were considered to be related to study drug. Dose reductions and/or treatment interruptions occurred more often with sorafenib than with regorafenib (Supplementary Table 1). Overall, in the majority of HCC-PDX models tested, significant TGI was observed with regorafenib and sorafenib treatment, with superior responses in the four models on treatment with regorafenib.

### Pharmacokinetics of regorafenib and sorafenib

To assess whether some of the differences in antitumor activity between regorafenib and sorafenib were due to differences in pharmacokinetics (PK), the PK profiles of the two drugs at steady state were determined in BALB/c nu/nu mice, the strain used for the HCC-PDX model. Formulations and doses were identical to the HCC-PDX study except that, for sorafenib, the dose was related to the tosylate salt (instead of the free base) to reflect internal standard procedures. For both drugs, major human metabolites (M-2 for sorafenib [13]; M-2 and M-5 for regorafenib [14]) were also determined. A previous study showed that mice metabolize both drugs similarly to humans [13, 14]. The cumulated exposure (sum of parent compound and metabolites) as assessed by the area under the curve during 24 hours at steady state AUC_(0–24)ss_ was 103,350 (μg·h)/L for regorafenib, after 10 mg/kg once daily for 5 consecutive days, and 83,560 (μg·h)/L for sorafenib, after 30 mg/kg once daily administration of sorafenib tosylate for 5 consecutive days, whereas an equal cumulated maximum concentration (C_max_) of 24.5 μMol/L was observed for both treatments (Table 2). With regard to the *in vivo* HCC-PDX study, in which the dose was related to the free base, the sorafenib exposure was estimated to be 114,516 (μg·h)/L in terms of AUC_(0–24)ss_ and 33.6 μMol/L in terms of C_max_, respectively, assuming dose linearity. As a result, it seems unlikely that the reduced activity seen with sorafenib in some models was due to lower exposure compared with regorafenib, thereby enabling comparison of antitumor data for regorafenib and sorafenib.

**Table 2:** Pharmacokinetic parameters of regorafenib and sorafenib and their metabolites M-2 and M-5 in BALB/c nu/nu mice after oral administration at doses of 10 mg/kg/day (regorafenib) and 30 mg/kg/day (sorafenib tosylate), respectively, for 5 consecutive days

Administered compound	Regorafenib			Sorafenib tosylate	
Analyte	Regorafenib^a^	M-2	M-5	Sorafenib^b^	M-2
**AUC**_**(0–24)ss**_ **[(μg·h)/L]**	93,100	9100	1150	82,400	1160
**AUC**_**(0–24)ss**_**[(μMol·h)/L]**	193	18.2	2.37	177	2.41
**AUC**_**(0–24)ss**_ **(percent of total)**^c^	90	9	1	99	1
**C**_**max**_ **[μg/L]**	10,700	1030	100	11,100	267
**C**_**max**_ **[μMol/L]**	22.2	2.06	0.21	23.9	0.56

^a^Regorafenib was administered in polypropylene glycol, PEG400, Pluronic F68, water (34:34:12:20). ^b^Sorafenib tosylate dose corresponds to 21.9 mg/kg/day of sorafenib and was administered in Cremophor, ethanol, water (12.5:12.5:75). ^c^Total refers to the sum of the molar concentrations of regorafenib (regorafenib, M-2, and M-5) or sorafenib (sorafenib and M-2), regardless of unbound fraction. AUC, area under the curve; C_max_, maximum concentration; PEG400, polyethylene glycol 400.

### Pharmacodynamic analysis of drug response by multiplex western blotting

**Figure 4:**
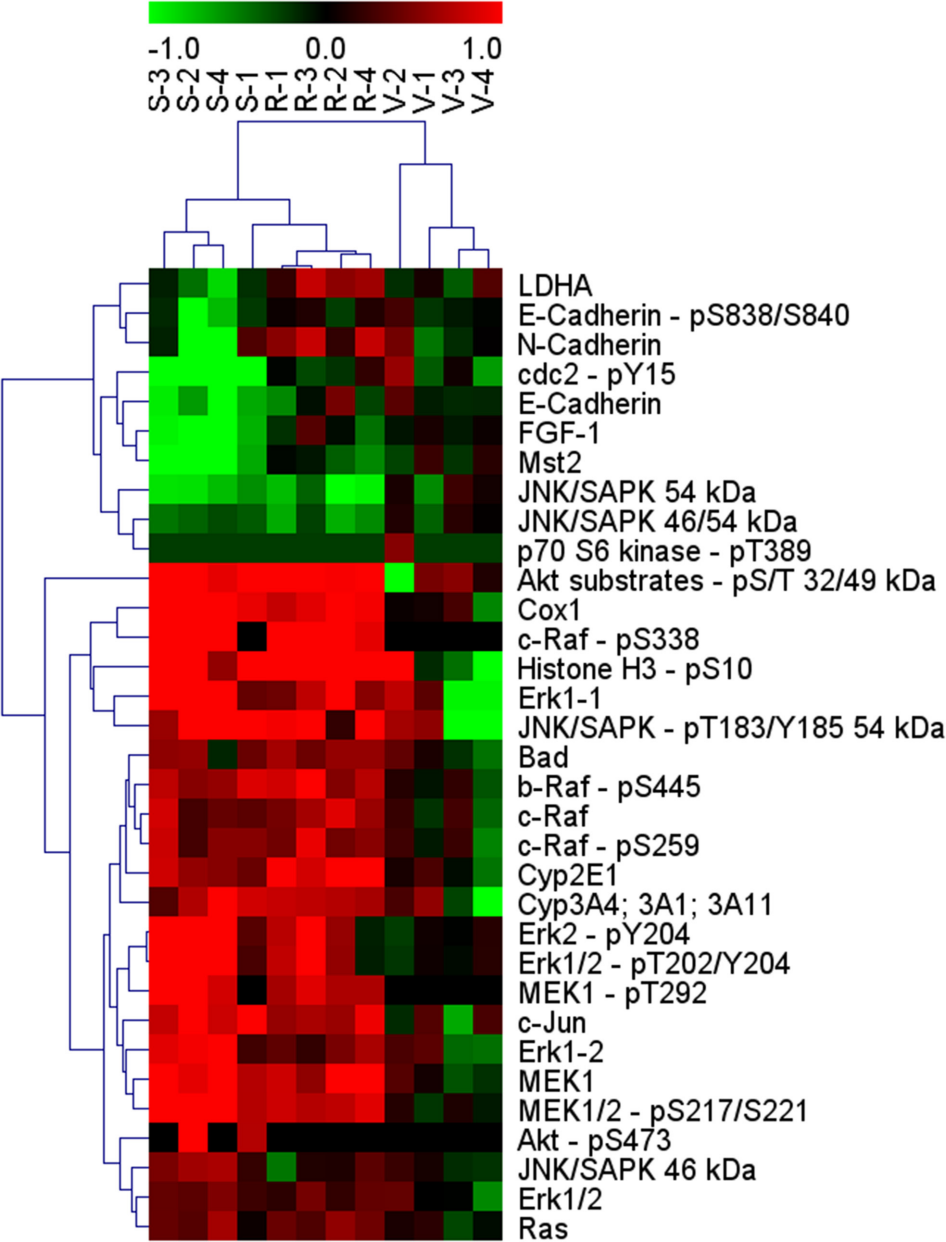
Effects of regorafenib or sorafenib treatment on selected protein analytes in HCC-PDX model 189 by a bead-based multiplex western blot analysis Proteins showing a significant change (p<0.05) in expression are displayed. For each treatment, four tumors from mice, which were not subject to dose reductions or dosing interruptions, were analyzed. Red, upregulated; green, downregulated. HCC-PDX, patient-derived hepatocellular carcinoma xenograft; R, regorafenib; S, sorafenib; V, vehicle.

To gain insight into the mechanism of action by which both drugs exert their antitumor effects, total protein lysates from drug- and vehicle-treated HCC-PDX model 189 xenografts were subjected to bead-based multiplex western blot analysis [15]. Model 189 was selected because all groups of this model were terminated at the same time, allowing for a comparative analysis between treatment groups. A total of 116 analytes were selected based on their previously described relationships to the mechanisms of action of regorafenib and sorafenib, including proteins involved in apoptosis, angiogenesis, proliferation, intracellular signaling, and metabolism. In this analysis, signals were detectable from 84 analytes; the change in their expression, compared with vehicle-treated samples, are displayed as a heat map in Supplementary Figure 1. Proteins with a significant change in expression (p<0.05), relative to the vehicle-treated samples, were identified and subjected to hierarchical clustering (Figure 4). The four samples of each treatment group cluster together. Patterns of protein expression in regorafenib- and sorafenib-treated samples were substantially different from those in the vehicle group. In drug-treated samples, more proteins appear upregulated than downregulated (red and green, respectively) (Figure 4). In addition, the pattern of upregulated proteins is similar among both drugs, whereas more proteins are downregulated in sorafenib- versus regorafenib-treated samples (Figure 4).

**Figure 5:**
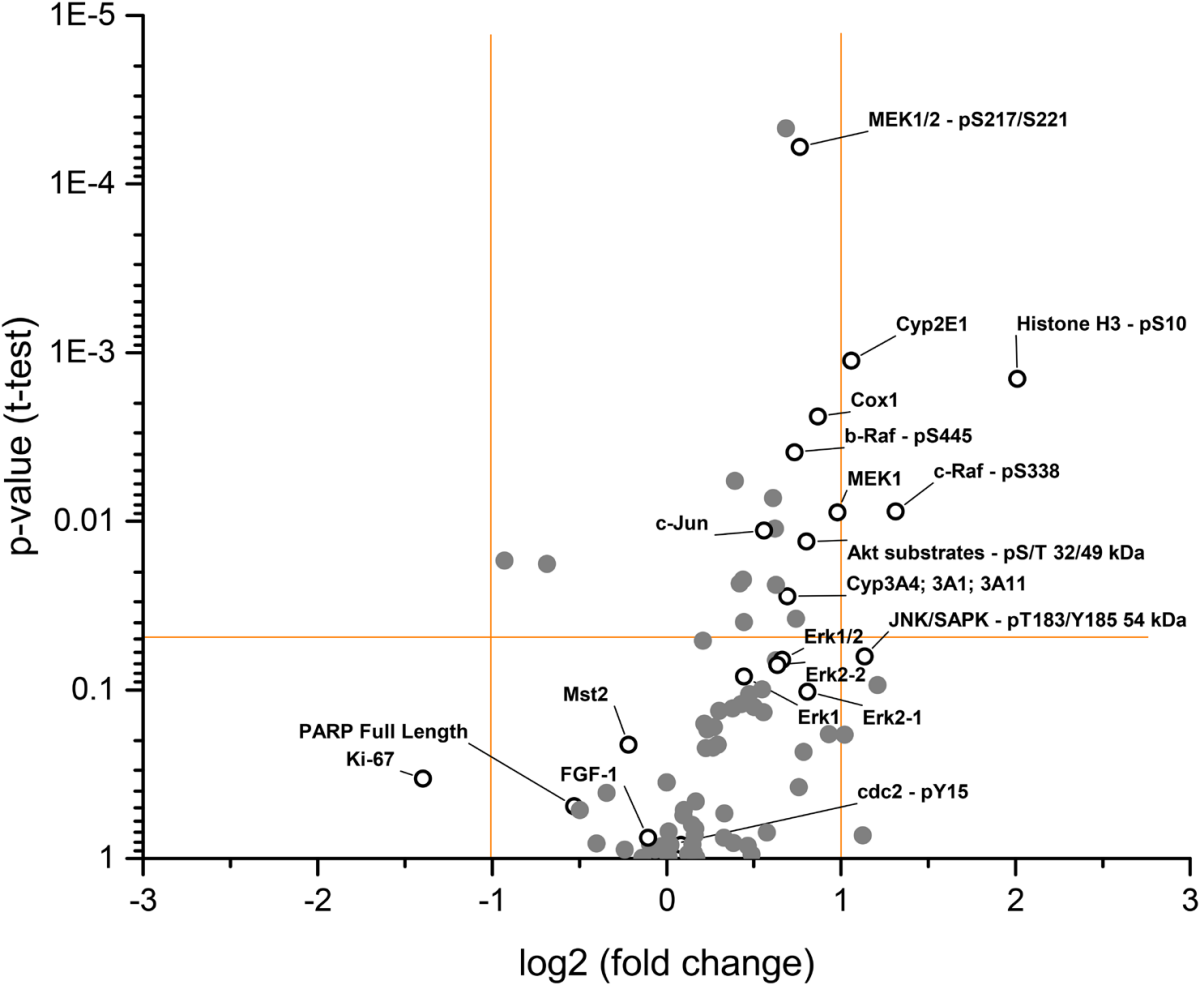

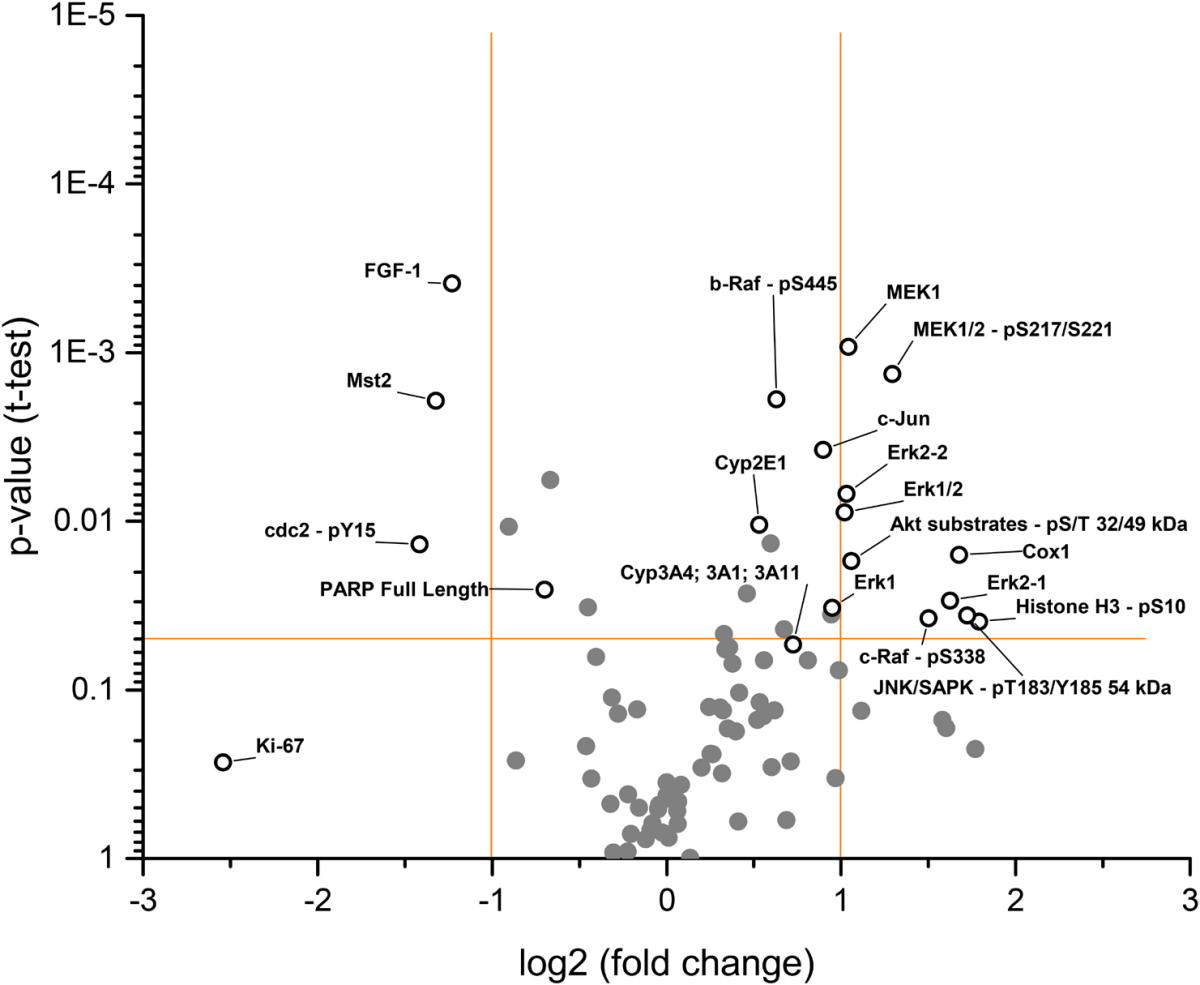
Fold changes of selected protein analytes (pooled) between **(A)** regorafenib and **(B)** sorafenib versus vehicle-treated HCC-PDX 189 xenografts. Components of interest are labeled with open circles and names. Data points above the horizontal orange bar are statistically significant (p<0.05). Vertical orange bars show the cut-offs for downregulation (fold change less than –1) and upregulation (fold change greater than 1). HCC-PDX, patient-derived hepatocellular carcinoma xenograft.

Interestingly, the RAS/RAF/MEK/ERK pathway, a major target of both drugs, appears activated, which is most evident by the significantly elevated levels of both phosphorylated and total MEK (MEK1/2-pS217/S221 and MEK1 in Figure 5) and, to a lesser degree, by the activation of other components of this pathway such as Raf and ERK (b-Raf-pS445, c-Raf-pS338, Erk1, and Erk2 in Figure 5). Elevated levels of AKT substrates and c-jun were observed, suggesting that other intracellular pathways may also be affected. Furthermore, elevated levels of Cox-1 (an inflammation marker) and, unexpectedly, pS10 histone H3 (a known proliferation marker) were observed. Minor effects on Ki67, another common proliferation marker, were found. Marginal but significant elevations of cytochrome P450 enzymes CYP3A4/1/11 (a known metabolizing enzyme of both compounds) and CYP2E1 were observed (Figure 5).

The major downregulated proteins with sorafenib were angiogenic fibroblast growth factor-1 (FGF-1), metabolic kinase Mst2 (which is biochemically not inhibited by both drugs), and cdc2-pY15 (a protein involved in cell cycle regulation) (Figure 5b). Full-length PARP, a marker for apoptosis, was also significantly downregulated, but to a lesser extent than the others mentioned. Taken together, the multiplex protein analysis of regorafenib- and sorafenib-treated tumor samples provides a new level of understanding as to the effects of these drugs on the tumor, and may serve as a starting point for establishing the difference between both drugs and for identifying the molecular basis for combination therapies in HCC.

## DISCUSSION

This study set out to assess the antitumor activity of regorafenib and sorafenib in various mouse models of HCC and to evaluate associated changes in the expression of functionally relevant proteins to gain insight into their mechanisms of action. In an orthotopic murine H129 liver tumor model, regorafenib significantly improved median survival versus vehicle, whereas sorafenib did not. Furthermore, in a set of 10 patient-derived HCC xenograft models implanted subcutaneously in mice, variable TGI was observed including both very good and very poor responders. Response was observed in the majority of models and was typically greater with regorafenib than sorafenib. Consistent with these results, inhibition of liver tumor models was previously observed for both drugs in a PLC/PRF/5 HCC model [16, 17] and for sorafenib in other HCC-PDX models [18, 19]. The better response reported by Gu et al. in models 10 and 20 is probably due to the higher dose of 60 mg/kg/day sorafenib used in their study [20]. Neither the general activity of both drugs nor the difference in activity between regorafenib and sorafenib observed in this study were found to be associated with any clinical characteristics published by Gu et al. [20] such as tumor stage, hepatitis B virus infection status, or genetic parameters (e.g. expression levels of alpha-fetoprotein or *TP53* mutation status); this lack of association may be attributed to the small sample size. Of note, the drugs also appeared to be active across fast- and slow-growing tumors (Figure 3).

Pharmacokinetic analyses indicate that the observed differences in activity between regorafenib and sorafenib, at least in the PDX models that were performed in the same BALB/c nude mouse strain as the PK study, are not associated with differences in exposure at the doses used. The syngenic orthotopic H129 model was grown in a different mouse strain (C3H/HeN) for which no PK analysis was performed, and therefore a PK effect cannot be excluded. Although we have previously observed different exposures at a given dose in different mouse strains [14], the relative exposures of both drugs remained similar (data not shown), which argues against different exposures of both compounds as an explanation for the different activities in the H129 model.

It should be mentioned that the binding of regorafenib, sorafenib, and metabolites to BALB/c nude murine plasma proteins was not analyzed in the present study. However, former studies indicated that the protein binding for all five compounds was high in CD-1 murine plasma (unbound fractions below 1%, individual values between 0.412 and 0.888) [13, 14].

Both treatments were generally well tolerated and no drug-related deaths occurred. However, dose reductions and/or treatment interruptions were more common with sorafenib than regorafenib, which could provide an explanation for the inferiority of sorafenib in some models. This explanation can be ruled out at least for model 10, which did not show differences in dose modifications between the two drugs. The reasons for the tolerability differences are unclear, but might relate to different drug metabolism in liver tumors, as observed for sorafenib [21], or possibly to potency differences in the inhibition of their target kinases [14, 22], which may also explain the efficacy of regorafenib in sorafenib-refractory patients in the recent phase 3 RESORCE trial [12]. However, because the HCC-PDX models used here were not derived from sorafenib-intolerant patients, it is currently unclear how well the HCC-PDX models that were less responsive to sorafenib reflect tumors that are responsive to regorafenib given after sorafenib.

To investigate further the mechanism by which regorafenib and sorafenib exert their antitumor effects, DigiWest, a new multiplex western blotting method, was used to analyze changes in protein expression upon treatment. DigiWest allowed the analysis of more than one hundred preselected proteins including markers for the proposed mechanism of action of both drugs in total lysates from entire xenografts of model 189 harvested 24 hours after the last drug application in a short time frame. While the pattern of significantly upregulated proteins was similar for regorafenib and sorafenib, there were differences in the pattern of proteins that were significantly downregulated, adding weight to the possibility that differences in mechanism of action might at least partially explain the activity of regorafenib in sorafenib-refractory patients [12]. Elevated levels of total and/or phosphorylated proteins within the RAS/RAF/MEK/ERK pathway were observed in regorafenib- and sorafenib-treated samples, suggesting activation of this pathway. This increase was unexpected considering that both drugs have known targets within this pathway. However, upregulation of the MAPK pathway has been previously observed in other HCC-PDX models treated with sorafenib, which was attributed at least in part to a concomitant elevation of IGFRß protein seemingly mediated via inhibition of p70S6K by sorafenib [19]. In the model reported here, no IGFRß increase or consistent p70S6K inhibition was observed (Supplementary Figure 1B), suggesting an involvement of other mechanisms such as transactivation after wild-type BRAF inhibition by sorafenib as recently described [23]. Notably, upregulation of MEK-ERK signaling has also been observed in biopsy samples of patients with colorectal cancer after regorafenib treatment, which however, did not correlate with progression-free survival [24]. The fact that tumor growth remained inhibited despite activation of this pathway suggests that it does not immediately trigger the initiation of proliferation, but may, accompanied by elevated pJNK, induce tumor cell quiescence [25]. Mechanistically, the growth inhibition of the PD xenografts appears to also be mediated by induction of apoptosis and by inhibition of proliferation as indicated by reductions of full-length poly (ADP-ribose) polymerase and Ki67, respectively, similar to previous observations [18, 19]. Compound-induced apoptosis is further supported by elevated levels of the pro-apoptotic protein BAD, whereas the anti-apoptotic protein Mcl-1 remained largely unaffected (Figure 4 and Supplementary Figure 1B). The reasons for the distinct and unexpected upregulation of pS10 histone H3, an occasionally used proliferation marker, are unclear, and this finding contrasts earlier reports [19, 26]. Histone H3 is also involved in transcriptional regulations [27] and its S10-phosphorylation may be stress induced [28]. Unfortunately, antibodies against total and phosphorylated VEGFR2 did not result in detectable signals in this assay, probably due to low VEGFR2 levels, precluding an assessment of antiangiogenic effects. In addition, no effects were observed on VEGF ligand levels. Notably, CYP3A4, a metabolizing enzyme of regorafenib and sorafenib, was upregulated by both compounds, possibly via a feedback loop. This is not unexpected since the induction of cytochrome P450 by xenobiotics, including small drug molecules, is a common phenomenon [29].

## MATERIALS AND METHODS

### Dose selection

Regorafenib and sorafenib tosylate were provided by Bayer AG, Germany. A regorafenib dose of 10 mg/kg/day and a sorafenib dose of 30 mg/kg/day was used, which lead to exposures in the range of the respective human dose of 160 mg/day [30] and 400 mg twice daily [5], respectively.

### Mouse experiments

All mouse experiments were approved by the relevant regulatory agency [(federal states of Nordrhein Westfalen, Landesamt für Natur, Umwelt und Verbraucherschutz, approval number 600/A02) and Berlin (Landesamt für Gesundheit und Soziales Berlin, approval number A0292/12)], and were conducted in compliance with the German Law of Animal Rights. Animals were kept in a 12-hour light / dark cycle, at a housing temperature of 23°C. Food and water was available *ad libitum*.

### Syngenic H129 mouse model

C3H/HeN mice aged 10–12 weeks with an average weight of 25 g were used for this model and were obtained from Charles River (Sulzfeld, Germany). Experiments were initiated after an acclimatizing period of at least 8 days. Syngenic murine H129 hepatoma cells (obtained from Dr. V. Schmitz, University of Bonn, Germany) were cultivated in RPMI medium, containing 10% fetal calf serum (FCS) and 5% glutamine. Cells were harvested in a subconfluent state (70–80%), prepared for injection of 5×10^4^ cells/20 μL in growth factor-free Matrigel, and transplanted in the left upper liver lobe. Animals with surgical failures were marked and excluded from the experiment. Treatment was initiated 4–5 days after transplantation. One set of mice received regorafenib 10 mg/kg once daily, vehicle solution once daily, or no treatment (n=8 per treatment group). A second set of mice received either sorafenib 30 mg/kg or vehicle solution once daily, or no treatment (n=8 per treatment group). Treatments were given orally via gavage. The vehicle solution contained 10% Transcutol, 10% Cremophor, and 80% 0.9% NaCl. Animals were weighed twice a week and monitored daily for behavior and wound healing. Animals were culled when they showed simultaneously labored breathing, impaired motility, and a low body temperature. Due to ascites formation in the peritoneum (a symptom of the disease), body weight was not used as part of the assessment. Animals were dissected to assess the cause of death unless they had died in their cage overnight.

### PDX mouse models

The PDX studies were carried out by WuXi AppTec (Shanghai, China). HCC-PDX models were originally established from surgically resected tumor tissue that was implanted in 6–8-week-old female BALB/c nude mice [20]. Animal studies were performed according to the guidelines approved by the Institutional Animal Care and Use Committee of Wuxi AppTec, which follow the guidance of the Association for the Assessment and Accreditation of Laboratory Animal Care. Animals were routinely monitored for their health status and their body weights. Treatment was suspended and/or doses reduced at body weight losses >10%. Animals were euthanized when the tumor size exceeded 2000 mm^3^. Pieces from tumor xenografts (∼30 mm^3^) from passages 4–8 (ST1) were implanted subcutaneously at the right flank, and tumors were grown to an average size of about 150–200 mm^3^. Mice were randomized into groups of 10 and treated by oral gavage once daily with: a) regorafenib at 10 mg/kg dissolved in polypropylene glycol, PEG400, Pluronic F68, water (34:34:12:20); b) sorafenib 30 mg/kg dissolved in Cremophor ethanol, water (12.5:12.5:75); or c) with an unspecific control murine IgG1 antibody (1B711) [31] dissolved in saline, which was given intraperitoneally at a dose of 25 mg/kg every other week and served as a control. This control was used because the study reported here was part of a larger investigation, and previous experiments demonstrated that various vehicles had no influence on tumor growth in comparison to non-treatment (Figure 1). Tumor size was measured twice weekly using caliper measurements. Tumor volume (mm^3^) was calculated using the formula 0.5 *a* × *b*^*2*^, where *a* and *b* are the long and short diameters of the tumor, respectively. TGI was assessed either by calculating the ratio of the mean tumor volumes of treated and control groups or by determination of the RTV, which relates tumor volumes at any given day of treatment to the volume at the first treatment day.

### Pharmacokinetic analyses of regorafenib and sorafenib

Pharmacokinetic studies were performed in 6–8-week-old female BALB/c nu/nu mice (Charles River, Sulzfeld, Germany). Regorafenib and sorafenib tosylate were each given to three animals per sampling time point, orally, once daily at a dose of 10 mg/kg and 30 mg/kg, respectively, for 5 days. The sorafenib tosylate dose corresponds to 21.9 mg/kg/day of sorafenib (free base). Parent compounds and metabolites M-2 and M-5 were analyzed in plasma samples using validated bioanalytical liquid chromatography-tandem mass spectrometry (LC-MS/MS) methods according to an experimental protocol described earlier [32]. PK parameters, namely the AUC_(0–24)ss_ and C_max_, were calculated from plasma concentrations using a non-compartmental analysis.

### Multiplex western blot analysis

For high-content Western analysis, DigiWest was performed as described by Treindl et al. [15]. In brief, xenograft tissue was ground by a Mikro-Dismembrator (Sartorius) and subsequently lysed by the addition of 2× LDS lysis buffer (Life Technologies^™^) and heated to 95°C for 9 minutes with several vortexing steps. Undissolved material was removed by consecutive centrifugation at 20°C for 2 minutes at 300 x g, 5 minutes at 16,000 x g, and through a QIAshredder homogenizer tube (Qiagen) for 5 minutes at 16,000 x g. Protein concentration was determined by in-gel staining. 3 μL lysate was separated by SDS-PAGE stained with IRDye^®^ Blue and detected with a Li-COR Odyssey Infrared Imaging System (Li-COR Biosciences, Bad Homburg, Germany) at 700 nm. Next, 12 μg protein per sample was separated using 4–12% Bis-Tris gels (Life Technologies™) according to the manufacturer’s instructions. Blotting onto PVDF membranes (Millipore) was performed under standard conditions. Proteins immobilized on the blotting membrane were biotinylated (NHS-PEG12-Biotin, Thermo Scientific) and individual sample lanes were cut into 96 strips each (height 0.5 mm each, strip width 7.5 mm) using an electronic cutting tool (Silhouette SD). Each individual strip was placed in a separate well of a 96-well plate, and protein was eluted for 2 hours in 10 μL elution buffer (8 M urea, 1% Triton-X100 in 100 mM Tris-HCl, pH 9.5). After the addition of 90 μL of dilution buffer (5% BSA in PBS, 0.02% sodium azide, 0.05% Tween-20), 96 different Neutravidin-coated Luminex bead sets (40,000 beads/well) were added to the individual wells, and eluted biotinylated proteins were captured on the bead surface during overnight incubation. After incubation, the Luminex beads were pooled, washed, and stored in storage buffer (1% BSA, 0.05% Tween-20, 0.05% sodium azide in PBS) at 4°C.

For antibody incubation, an aliquot of the bead pool was transferred into an assay plate and 30 μL of diluted western blot antibody in assay buffer (Blocking Reagent, Roche Applied Science; 0.05% Tween 20, 0.02% sodium azide, 0.2% milk powder) was added per well. 116 antibody incubations on 12 samples were performed overnight at 4°C. For read-out, beads were washed twice with 100 μL of PBST before species-specific PE-labeled secondary antibodies (Jackson) were added in 30 μL of assay buffer for 1 hour. After two washes with 100 μL of PBST, the signal was generated in a FlexMAP 3D instrument (Luminex).

### Data analysis

DigiWest analysis was performed as previously described [15]. In brief, data generated by the Luminex instrument were analyzed using a dedicated analysis tool that visualizes the fluorescent signals as bar graphs and identifies antibody-specific peaks. Each graph is composed of the 96 values derived from the 96 molecular weight fractions obtained after antibody incubation. The software tool identifies specific peaks and a molecular weight is assigned to each of the 96 fractions. After background correction, specific signal intensities are calculated as the integral of the identified peak.

### Statistical analysis

In the syngenic orthotopic H129 model, differences in survival times between groups were analyzed for significance using log-rank tests. Analysis of tumor volumes of the HCC-PDX models was performed with log_2_-transformed values on defined days of measurement based on the assumption of their log-normal distribution. Log_2_-transformed tumor volumes of regorafenib and sorafenib versus their mutual vehicle and versus each other were evaluated. All respective statistical test results were derived from a common linear model that allows for individual variances in the different groups. The adjustment for multiple testing was carried out using Šidák correction at a significance level of 0.05. Back transformation of the mean difference of log_2_-transformed tumor volumes led to the ratios of the compared groups, whereby ratios of 1 reflect no difference in tumor volumes and ratios below or above 1 reflect smaller or larger tumor volumes, respectively, of the respective second group. The analyses of RTV were carried out in a similar fashion without the log_2_ transformation of values. Bead-based western data were analyzed (described in [15]) using the MEV 4.8.1 software package [33]. A p-value <0.05 was regarded as significant. All statistical analyses were performed with SAS^®^ 9.4 [34].

## CONCLUSIONS

The present data suggest that although both regorafenib and sorafenib are effective in preclinical HCC models, there appears to be substantial differences in their mechanisms of action, as evidenced by differences in their antitumor activities in preclinical models and in their effects on protein expression. These data may explain the efficacy of regorafenib in sorafenib-refractory patients in the phase 3 RESORCE trial.

## SUPPLEMENTARY MATERIALS FIGURE AND TABLES







## Abbreviations

AUC_(0–24)ss_: area under the curve during 24 hours at steady state
CI: confidence interval
C_max_: maximum concentration
FCS: fetal calf serum
HCC: hepatocellular carcinoma
HR: hazard ratio
LC-MS/MS: liquid chromatography-tandem mass spectrometry
MKI: multikinase inhibitor
OS: overall survival
PK: pharmacokinetic
PDGFR: platelet-derived growth factor receptor
PDX: patient-derived xenograft
RT: room temperature
RTV: relative tumor volume
T/C: treated vs control (vehicle) ratio
TGI: tumor growth inhibition
VEGFR: vascular endothelial growth factor receptor
