# Supplementary material for: Antitumor effects of regorafenib and sorafenib in preclinical models of hepatocellular carcinoma
Source: Oncotarget. 2017 Nov 6;8(63):107096–108. doi: 10.18632/oncotarget.22334 (PMC5739799; doi:10.18632/oncotarget.22334)
Supplement: Supplementary file 1 [file oncotarget-08-107096-s001.pdf]

Antitumor effects of regorafenib and sorafenib in preclinical models of hepatocellular carcinoma

SUPPLEMENTARY MATERIALS

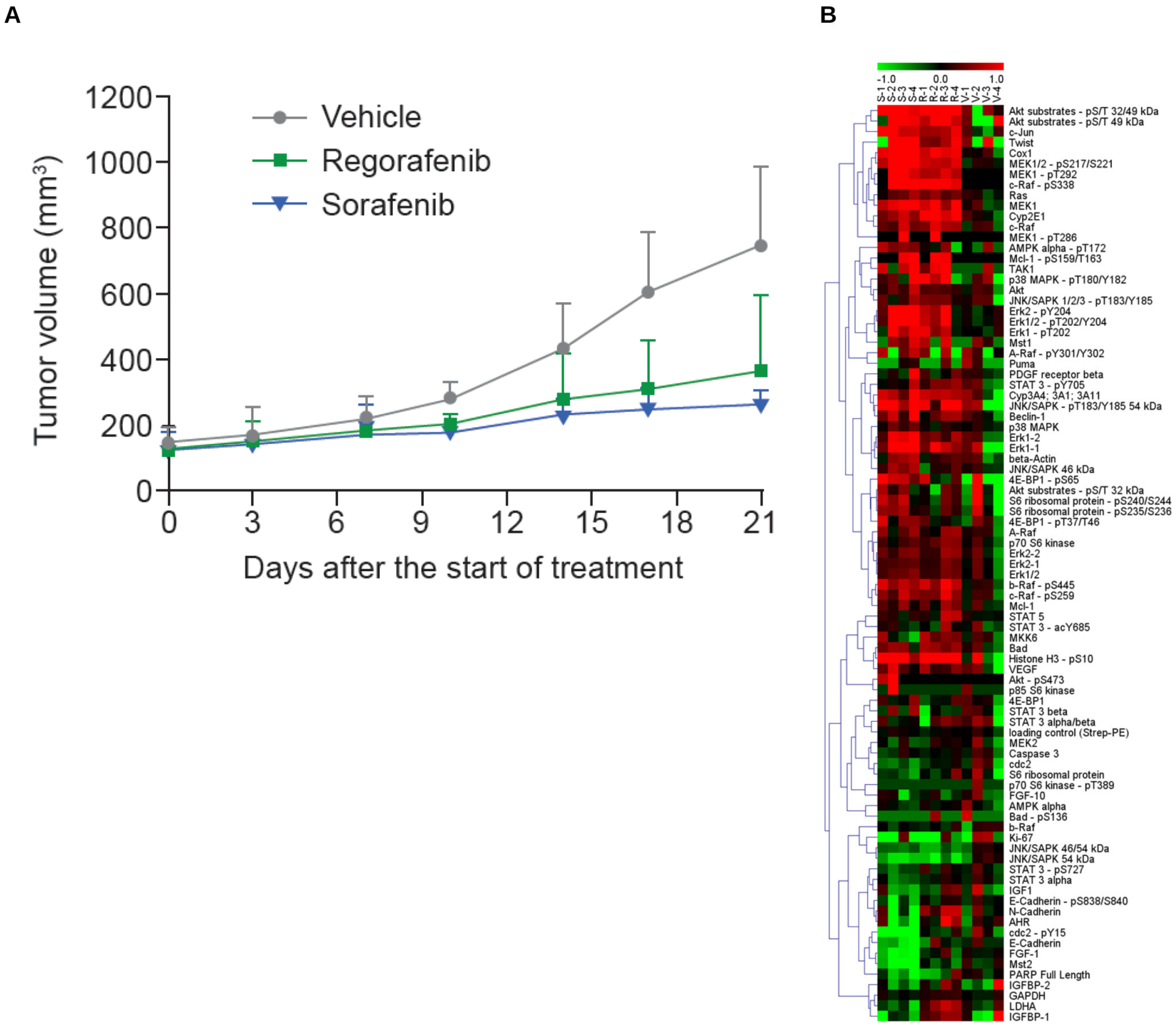

Supplementary Figure 1: Effects of regorafenib/sorafenib treatment on selected protein analytes in HCC-PDX model 189. **(A)** Growth curves of the four tumors of each treatment used for the analysis; **(B)** Heat map of the expression changes of all selected protein targets analyzed. Error bars indicate standard deviations. HCC-PDX, patient-derived hepatocellular carcinoma xenograft; R, regorafenib; S, sorafenib; V, vehicle. Four samples were analyzed for each treatment. Red, upregulated; green, downregulated.

Supplementary Table 1: Summary of the results of the HCC-PDX models

See Supplementary File 1

Supplementary Table 2: Statistical analyses of tumor volumes at the end of the treatment period and of relative tumor volumes at the end of study

See Supplementary File 2
