# Supplementary material for: Antitumor effects of regorafenib and sorafenib in preclinical models of hepatocellular carcinoma
Source: Oncotarget. 2017 Nov 6;8(63):107096–108. doi: 10.18632/oncotarget.22334 (PMC5739799; doi:10.18632/oncotarget.22334)
Supplement: Supplementary file 2 [file oncotarget-08-107096-s002.docx]

**Supplementary Table 1: Summary of the results of the HCC-PDX models**

| Model | 5 | | 10 | 19 | 20 | 61 | 101 | 141 | 159 | 189 | 217 |
| --- | --- | --- | --- | --- | --- | --- | --- | --- | --- | --- | --- |
| Passage | | **5** | **7** | **9** | **7** | **8** | **5** | **5** | **5** | **5** | **4** |
| Sorafenib | | | | | | | | | | | |
| Starting dose [mg/kg/day] | | 30 | 30 | 30 | 30 | 30 | 30 | 30 | 30 | 30 | 30 |
| Duration of treatment [day] | | 28 | V:12; C:28 | 28 | 28 | 25 | 28 | 28 | 28 | 21 | V:13; C:20 |
| Duration of study [day] | | 46 | V:12; C:30 | 42 | 42 | V:32; C:36 | 42 | 42 | 35 | 21 | V:13; C:20 |
| Reduced dose [mg/kg/day]: days after treatment start | | 15:12-20; 7,5:21-28 | - | 15:11-28 | - | - | 15:10-14; 7,5:15-28 | 15:13-28 | 15:11-28 | 15:21-22 | - |
| Treatment suspension [n:d] | | - | - | 2:11-14/19 | - | 9:5-25 variable | - | 1:11-12/15-17 | 2:15-20 | 5:7; 1:17 | - |
| Mean TV | | 134.44 | 607.16 | 496.54 | 737.74 | 424.86 | 416.97 | 139.51 | 1144.22 | 351.96 | 1087.45 |
| TV/SD | | 100.08 | 207.87 | 208.17 | 222.95 | 144.78 | 192.13 | 119.99 | 340.76 | 105.96 | 538.90 |
| RTV | | 1.74 | 13.39 | 4.70 | 10.55 | 5.28 | 5.37 | 2.16 | 9.73 | 2.66 | 10.84 |
| RTV/SD | | 1.41 | 7.14 | 1.69 | 4.12 | 1.55 | 2.35 | 1.11 | 3.40 | 0.77 | 2.14 |
|  | |  |  |  |  |  |  |  |  |  |  |
| Regorafenib | | | | | | | | | | | |
| Starting dose [mg] | | 10 | 10 | 10 | 10 | 10 | 10 | 10 | 10 | 10 | 10 |
| Duration of treatment [day] | | 28 | V:12; C:28 | 28 | 28 | 28 | 28 | 28 | 28 | 21 | V:13; C:24 |
| Duration of study [day] | | 46 | V:12; C:40 | 42 | 42 | V:32; C:42 | 42 | 42 | 35 | 21 | V:13; C:24 |
| Reduced dose [mg/kg/day]: treatment days | | 7,5:12-28 | - | - | - | - | - | 7,5:13-28 | - | - | - |
| Treatment suspension [n:day] | | - | - | - | - | - | - | - | - | 1:21 | - |
| Mean TV | | 80.96 | 332.11 | 542.38 | 742.98 | 331.96 | 177.21 | 89.74 | 864.43 | 322.88 | 932.43 |
| TV/SD | | 48.91 | 148.26 | 299.10 | 325.19 | 128.09 | 102.04 | 48.55 | 175.35 | 150.96 | 252.38 |
| RTV | | 1.14 | 9.15 | 5.21 | 9.46 | 5.43 | 2.06 | 1.04 | 9.04 | 2.39 | 12.77 |
| RTV/SD | | 0.70 | 5.92 | 1.40 | 6.63 | 2.44 | 1.09 | 0.78 | 2.15 | 0.95 | 4.96 |

Days after treatment start are given, whereby the first day of treatment is referred to as Day 0. Duration of study indicates the last day on which a measurement was made. TV was calculated either at the end of the vehicle group or at the end of treatment if the vehicle group was still viable (the exception was model 20, in which Day 21 was used due to animal deaths in the vehicle group). RTV was calculated at the last day on which the regorafenib and sorafenib groups were still viable, independent of treatment cessation. C, compound; d, day; HCC-PDX, patient-derived hepatocellular carcinoma xenograft; n, number; RTV, relative tumor volume; SD, standard deviation; TGI, tumor growth inhibition; TV, tumor volume; V, vehicle.
