# Supplementary material for: Antitumor effects of regorafenib and sorafenib in preclinical models of hepatocellular carcinoma
Source: Oncotarget. 2017 Nov 6;8(63):107096–108. doi: 10.18632/oncotarget.22334 (PMC5739799; doi:10.18632/oncotarget.22334)
Supplement: Supplementary file 3 [file oncotarget-08-107096-s003.docx]

**Supplementary Table 2: Statistical analyses of tumor volumes at the end of the treatment period and of relative tumor volumes at the end of study**

| Study | Treatment group | Day of TV analysis^1^ | Day of RTV analysis^2^ | Comparison | | log TV | | | | RTV | | |
| --- | --- | --- | --- | --- | --- | --- | --- | --- | --- | --- | --- | --- |
|  |  |  |  |  |  | **Differences of least square-means** | **Ratio^3^** | **Standard error** | **Adj  p-value** | **Differences of least square-means** | **Standard error** | **Adj  p-value** |
| 5 | V | 28 (9) | 46 (8) | R | S | -0.4634 | 0.73 | 0.6073 | 0.8357 | -0.6001 | 0.4981 | 0.5602 |
|  | R | 28 | 46 | R | V | -29,518 | 0.13 | 0.4380 | **<.0001** | -60,049 | 11,660 | **<.0001** |
|  | S | 28 | 46 | S | V | -24,884 | 0.18 | 0.5891 | **0.0008** | -54,048 | 12,290 | **0.0005** |
| 10 | V | 12 | 12 | R | S | -0.9111 | 0.53 | 0.2378 | **0.0021** | -99,327 | 24,470 | **0.0008** |
|  | R | 12 | 40 | R | V | -27,077 | 0.15 | 0.3233 | **<.0001** | n.d. | |  |
|  | S | 12 | 30 (9) | S | V | -17,966 | 0.29 | 0.3039 | **<.0001** | n.d. | |  |
| 19 | V | 28 | 42 (9) | R | S | 0.08047 | 1.06 | 0.3274 | 0.9929 | 0.5174 | 0.7177 | 0.8575 |
|  | R | 28 | 42 | R | V | -0.6083 | 0.66 | 0.2862 | 0.1242 | -24,098 | 0.8745 | **0.0320** |
|  | S | 28 | 42 | S | V | -0.6887 | 0.62 | 0.2795 | 0.0607 | -29,272 | 0.9409 | **0.0138** |
| 20 | V | 21 | 42 (5) | R | S | -0.1048 | 0.93 | 0.3032 | 0.9808 | -10,984 | 24,697 | 0.9610 |
|  | R | 21 | 42 | R | V | -0.4621 | 0.73 | 0.4194 | 0.6272 | -23,207 | 24,854 | 0.7386 |
|  | S | 21 | 42 | S | V | -0.3573 | 0.78 | 0.3566 | 0.6927 | -12,223 | 18,639 | 0.8886 |
| 61 | V | 25 (9) | 32 (9) | R | S | -0.4771 | 0.72 | 0.3220 | 0.3869 | -19,538 | 0.6523 | **0.0081** |
|  | R | 25 | 42 (9) | R | V | -19,194 | 0.26 | 0.4492 | **0.0007** | n.d. | |  |
|  | S | 25 | 36 | S | V | -14,423 | 0.37 | 0.3659 | **0.0016** | n.d. | |  |
| 101 | V | 28 | 42 (8) | R | S | -12,850 | 0.41 | 0.3385 | **0.0023** | -33,185 | 0.8205 | **0.0013** |
|  | R | 28 | 42 | R | V | -24,002 | 0.19 | 0.4054 | **<.0001** | -54,553 | 0.9286 | **<.0001** |
|  | S | 28 | 42 | S | V | -11,152 | 0.46 | 0.3894 | **0.0238** | -21,367 | 11,390 | 0.2018 |
| 141 | V | 28 | 42 | R | S | -0.3980 | 0.76 | 0.5063 | 0.8233 | -11,236 | 0.4364 | **0.0474** |
|  | R | 28 | 42 | R | V | -33,363 | 0.10 | 0.3271 | **<.0001** | -66,708 | 11,034 | **<.0001** |
|  | S | 28 | 42 | S | V | -29,383 | 0.13 | 0.4189 | **<.0001** | -55,472 | 11,283 | **0.0001** |
| 159 | V | 28 | 35 | R | S | -0.3604 | 0.78 | 0.1746 | 0.1393 | -0.6894 | 12,719 | 0.9322 |
|  | R | 28 | 35 | R | V | -0.7546 | 0.59 | 0.1672 | **0.0003** | -32,205 | 13,405 | 0.0687 |
|  | S | 28 | 35 | S | V | -0.3943 | 0.76 | 0.2109 | 0.2020 | -25,310 | 15,768 | 0.3187 |
| 189 | V | 21 | 21 | R | S | -0.1791 | 0.88 | 0.2229 | 0.8135 | -0.2628 | 0.3879 | 0.8778 |
|  | R | 21 | 21 | R | V | -12,395 | 0.42 | 0.2941 | **0.0008** | -31,656 | 0.6077 | **<.0001** |
|  | S | 21 | 21 | S | V | -10,604 | 0.48 | 0.2681 | **0.0015** | -29,028 | 0.5817 | **<.0001** |
| 217 | V | 13 | 13 | R | S | -0.1276 | 0.92 | 0.2347 | 0.9316 | -14,902 | 12,570 | 0.2512 |
|  | R | 13 | 24 | R | V | -11,395 | 0.45 | 0.1975 | **<.0001** | n.d. | |  |
|  | S | 13 | 20 | S | V | -10,119 | 0.50 | 0.2594 | **0.0017** | n.d. | |  |

Significance statements are derived from ‘adj p-value’; significant values p<0.05 are in bold. ^1^Day post treatment start of statistical TV analysis; ^2^Day post treatment start of statistical (TV) analysis; ^1,2^Number in brackets indicates the number of animals that remain from a group of 10; ^3^‘Ratio’ refers to respective comparator ratios. n.d., not done; R, regorafenib; RTV, relative tumor volume; S, sorafenib; TV, tumor volume; V, vehicle.
